# Presenting features and long-term effects of growth hormone treatment of children with optic nerve hypoplasia/septo-optic dysplasia

**DOI:** 10.1186/1687-9856-2011-17

**Published:** 2011-11-15

**Authors:** Amy M Vedin, Hanna Karlsson, Cassandra Fink, Mark Borchert, Mitchell E Geffner

**Affiliations:** 1 Center for Endocrinology, Diabetes, and Metabolism, Children's Hospital Los Angeles, 4650 Sunset Boulevard, Mailstop #61, Los Angeles, CA 90027, USA; 2 Pfizer Inc., Pfizer Endocrine Care, KIGS/KIMS/ACROSTUDY, SE-191 90 Sollentuna, Sweden; 3 The Vision Center, Children's Hospital Los Angeles, 4650 Sunset Boulevard, Mailstop #88, Los Angeles, CA 90027, USA; 4 Saban Research Institute of Children's Hospital Los Angeles, 4650 Sunset Boulevard, Los Angeles, CA 90027, USA

## Abstract

**Background:**

Optic nerve hypoplasia (ONH) with/or without septo-optic dysplasia (SOD) is a known concomitant of congenital growth hormone deficiency (CGHD).

**Methods:**

Demographic and longitudinal data from KIGS, the Pfizer International Growth Database, were compared between 395 subjects with ONH/SOD and CGHD and 158 controls with CGHD without midline pathology.

**Results:**

ONH/SOD subjects had higher birth length/weight, and mid-parental height SDS. At GH start, height, weight, and BMI SDS were higher in the ONH/SOD group. After 1 year of GH, both groups showed similar changes in height SDS, while weight and BMI SDS remained higher in the ONH/SOD group. The initial height responses of the two groups were similar to those predicted using the KIGS-derived prediction model for children with idiopathic GHD. At near-adult height, ONH/SOD and controls had similar height, weight, and BMI SDS.

**Conclusions:**

Compared to children with CGHD without midline defects, those with ONH/SOD presented with greater height, weight, and BMI SDS. These differences persisted at 1 year of GH therapy, but appeared to be overcome by long-term GH treatment.

## Background

Optic nerve hypoplasia (ONH) is a congenital anomaly often associated with hypopituitarism and brain malformations. It is relatively rare, with an incidence of 1 in 10,000 live births, and it equally affects boys and girls [1]. The term septo-optic dysplasia (SOD), historically and even today, is widely used interchangeably with that of ONH. However, it is now known that absence of the septum pellucidum *per se *does not confer increased risk for growth hormone (GH) deficiency alone or as a component of hypopituitarism in children with ONH [2,3].

In a study of 47 subjects [age (mean ± SD) 15.2 ± 10.6 months] with ONH followed until 59.0 ± 6.2 months of age, Ahmad, *et al *reported a prevalence of endocrinopathies of 71.7% (including 64.1% with GH-IGF-I axis abnormalities); these were not associated with ONH laterality, absence of septum pellucidum, or pituitary abnormalities on neuro-imaging [2]. The only prior large, long-term study looking at growth outcomes in children described as having SOD treated with GH included 582 children enrolled in the National Cooperative Growth Study (NCGS) (Genentech, S. San Francisco CA). Among this cohort, 71 reached near-adult height (NAH) (mean -1.57 ± 1.27 SD), representing a mean gain of 1.17 ± 1.49 SD after 6-7 years of GH treatment [4].

More recently, it has been suggested that obesity is a frequent occurrence in children with ONH perhaps on a hypothalamic basis [5,6]. In his cohort of 47 subjects with ONH, Ahmad found that 44% had a body mass index (BMI) > 85^th ^percentile at 5 years of age [2]. In the large NCGS study, no weight or BMI data were reported [4].

The purpose of the current analyses is to compare presenting features and short- and long-term auxological outcomes of GH treatment in children with ONH to those of patients with congenital growth hormone deficiency (CGHD) without non-pituitary midline defects or ONH using data from KIGS (the Pfizer International Growth Database).

## Methods

The KIGS database, established in 1987 and containing data from over 70,000 patients in 51 different countries, is an international registry developed with the main objective of documenting the long-term outcomes and safety of Somatonorm^® ^and Genotropin^® ^GH products (Pfizer, Inc). The KIGS survey is performed in accordance with the recommendations adopted by the 18^th ^World Medical Assembly (held in Helsinki, Finland in 1964) and any subsequent revisions which exist to guide physicians carrying out biomedical research involving human individuals. Each subject and/or his/her legal representative receive adequate information, has the right to withdraw from the survey at any time, and consents his/her participation, although, during the first decade of its existence, this kind of registry or non-interventional trial that KIGS represents did not require informed consent from the subjects or legally acceptable representatives in many countries.

To capture subject data from KIGS for the current investigation, we included the diagnoses, ONH *and/or *SOD, since the latter is still widely used to describe patients with ONH whether or not the presence of the septum pellucidum is documented. Hereafter, for simplicity, we refer to the study condition as ONH/SOD. As of January 2009, there were 565 subjects identified as having CGHD secondary to ONH/SOD and 244 control subjects with CGHD unrelated to ONH/SOD and without extra-pituitary midline pathology. A list of the diagnostic codes included in the non-ONH/SOD CGHD group is included in Table 1. Subjects that were prepubertal and had at least one year of longitudinal data while receiving GH were included in the data analysis (ONH/SOD group n = 395 and CGHD group n = 158).

**Table 1:** KIGS diagnostic codes included in non-ONH/SOD CGHD (cross-sectional numbers)

KIGS Diagnostic Codes	n	Description
2.1.1.1	23	*GH *gene-defect (Type 1A dominant or recessive)
2.1.1.2	25	*GH *gene-defect
2.1.1.3	4	*GHRH *gene-defect
2.1.1.9	192	Other genetic cause of GHD

**Total**	**244**	

GH, growth hormone; GHRH, growth hormone releasing hormone; GHD, growth hormone deficiency

Background demographic characteristics, birth measurements, GH stimulation test results, and prevalence of associated hypothalamic-pituitary deficiencies affecting thyroid function, glucocorticoid production, and water metabolism (diabetes insipidus) were collected from KIGS. The prevalence of hypogonadism could only be obtained in older subjects who reached NAH. The subjects' additional hormonal deficiencies were also managed by their treating physicians. Auxological data at the time of initiation of GH therapy and after one year of treatment were also collected. Similar data of the subsets of the two groups who attained NAH were compared. NAH was defined by height velocity (< 2 cm/year), bone age (≥ 14 years in females or ≥ 16 years in males), and/or chronological age (> 15 years in females and > 17 years in males).

Growth parameters are reported as standard deviation scores (SDS) which were calculated based on standards from Prader *et al *[7] and, for weight data, from Freeman *et al *[8]. Birth weight and length SDS were calculated using the reference of Niklasson *et al *[9]. Bone age readings were taken as reported by physicians and were based on the methods of Greulich and Pyle [10] or Tanner *et al *[11,12]. Data were not normally distributed and, therefore, are presented as median and 10^th ^and 90^th ^percentiles. Wilcoxon rank-sum test was used to detect differences between the two groups.

The heights of subjects in both groups were analyzed using the previously published KIGS prediction model for idiopathic GHD excluding GH maximum peak [13]. Differences between observed and predicted height velocities are expressed in terms of Studentized residuals. The residual is calculated as the observed height velocity minus the predicted height velocity for each observation, and the Studentized residual is the residual divided by its standard error.

## Results

Background characteristics were compared between the two groups (Table 2). The birth length and weight SDS were significantly greater in the ONH/SOD group compared to the non-ONH/SOD CGHD group [birth length SDS median (0.27); 10^th ^and 90^th ^percentiles (-1.3, 2.0) vs. median (-0.62); 10^th ^and 90^th ^percentiles (-2.5, 1.2); p < 0.001) and birth weight SDS (-0.31; -1.7, 1.1) vs. (-0.58; -2.0, 1.0); p = 0.021)]. Mid-parental height SDS was also significantly greater in the ONH/SOD group (-0.23; -1.8, 1.3) compared to the non-ONH/SOD CGHD group [(-1.04; -3.0, 0.5); p < 0.001]. Peak GH levels on stimulation testing were similar between the two groups. The ONH/SOD group had significantly more subjects with other hypothalamic-pituitary hormone deficiencies compared to the non-ONH/SOD CGHD group (72.7% vs. 51.9%; p < 0.001), with involvement of the thyroid and adrenal axes being the most common.

**Table 2:** Characteristics at background, GH start, and 1^st ^year on GH

	ONH/SOD			Non-ONH/SOD			
	N	Median	10^th^, 90^th ^percentiles	N	Median	10^th^, 90^th ^percentiles	P-value
	*Background*						
Gender, males	395	227 (57.5%)		158	101 (63.9%)		
Birth Length SDS	206	0.27	-1.3, 2.0	104	-0.62	-2.5, 1.2	< 0.001
Birth Weight SDS	349	-0.31	-1.7, 1.1	133	-0.58	-2.0, 1.0	0.021
Mid-Parental Height SDS	324	-0.23	-1.8, 1.3	140	-1.04	-3.0, 0.5	< 0.001
Peak GH (μg/L)	307	2.60	0.8, 7.2	131	3.30	0.5, 10.8	0.053
Other Pituitary Hormone Deficiencies	395	287 (72.7%)		158	82 (51.9%)		< 0.001
Hypothyroidism	395	250 (63.3%)		158	74 (46.8%)		< 0.001
Glucocorticoid deficiency	395	206 (52.2%)		158	46 (29.1%)		< 0.001
Diabetes insipidus	395	51 (12.9%)		158	6 (3.8%)		< 0.001
	*At GH Start*						
Age (years)	395	3.99	0.9, 9.8	158	4.47	0.7, 10.7	0.498
Bone Age (years)	77	3.50	1.4, 8.7	41	4.00	1.2, 10.0	0.784
Height SDS	395	-3.00	-4.8, -0.9	158	-3.68	-6.7, -1.9	< 0.001
Height - Mid-Parental Height SDS	324	-2.74	-4.67, -0.62	140	-2.42	-4.94, -0.72	0.305
Height Velocity (cm/year)	147	5.46	2.35, 12.07	45	5.23	3.19, 9.76	0.904
Weight SDS	395	-1.56	-4.2, 0.5	158	-2.56	-5.7, -0.6	< 0.001
BMI SDS	395	0.22	-1.8, 2.1	158	-0.18	-2.1, 1.6	0.001
GH Dose (mg/kg/week)	395	*0*.24	0.16, 0.33	158	0.22	0.16, 0.39	0.395
	*1st yr on GH*						
Age (years)	395	5.01	1.87, 10.74	158	5.46	1.64, 11.75	0.500
Bone Age (years)	96	4.40	2.00, 9.00	53	4.17	2.00, 11.5	0.429
Height SDS	395	-1.95	-4.0, 0.0	158	-2.37	-4.7, -0.9	< 0.001
Δ Height SDS	395	1.02	0.0, 2.0	158	1.10	0.3, 2.7	0.066
Height - Mid-Parental Height SDS	324	-1.70	-3.78, 0.29	140	-1.29	-3.15, 0.23	0.010
Height Velocity (cm/year)	395	11.25	6.74, 16.18	158	10.54	6.98, 18.97	0.863
Weight SDS	393	-0.91	-3.3, 1.3	158	-1.81	-3.9, -0.0	< 0.001
BMI SDS	393	0.05	-1.8, 2.3	158	-0.43	-2.1, 1.2	< 0.001
GH Dose (mg/kg/week)	388	0.23	0.14, 0.33	156	0.21	0.13, 0.33	0.068
	*IGHD Prediction Model (Without GH Peak) at 1 year*						
Predicted Height Velocity	186	10.69	9.03, 12.51	73	9.99	8.28, 11.86	< 0.001
Actual Height Velocity (cm/year)	186	10.72	6.66, 14.37	73	9.49	7.16, 13.77	0.305
Studentized Residual	186	-0.11	-2.53, 1.98	73	-0.04	-1.61, 1.95	0.565

SDS, standard deviation score; GH, growth hormone

At the start of GH therapy, both groups had similar chronological ages and bone ages. However, the ONH/SOD group had significantly greater height SDS [(-3.00; -4.8, -0.9) vs. (-3.68; -6.7, -1.9); p < 0.001], weight SDS [(-1.56; -4.2, 0.5) vs. (-2.56; -5.7, -0.6); p < 0.001], and BMI SDS [(0.22; -1.8, 2.1) vs. (-0.18; -2.1, 1.6); p = 0.001] (Table 2).

**Figure 1:**
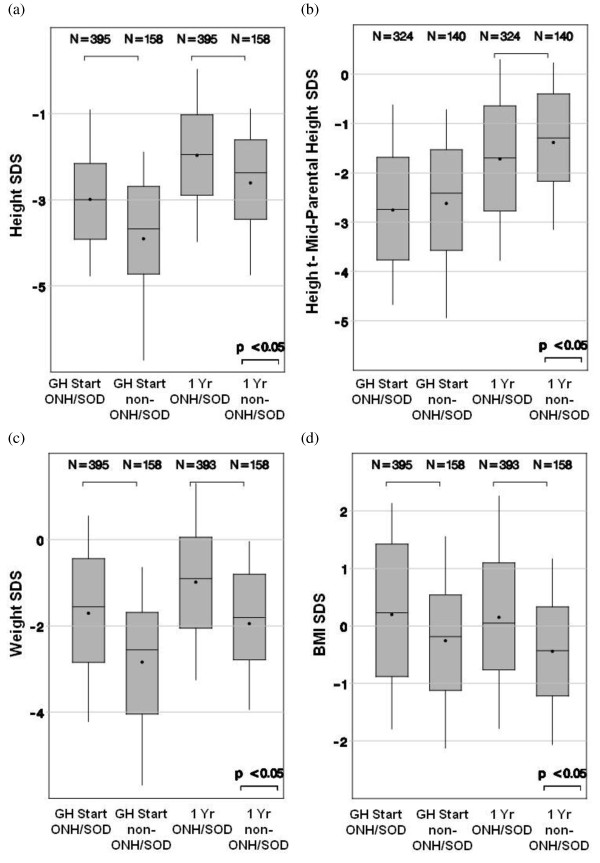
**Comparison between groups**. Comparison between groups with ONH/SOD vs. non-ONH/SOD CGHD of (a) height SDS, (b) height - MPH SDS, (c) weight SDS, and (d) BMI SDS at start and after 1 year of GH therapy.

**Figure 2:**
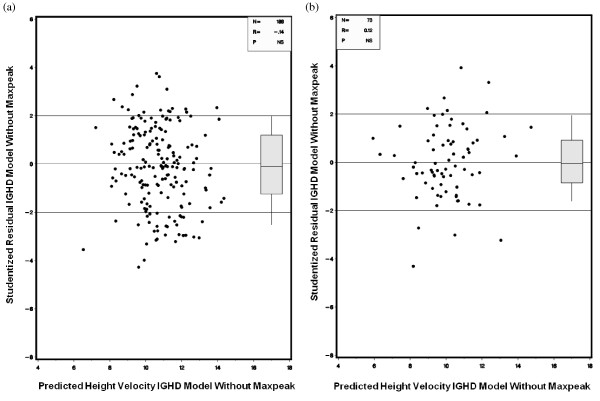
**Differences between observed and predicted height velocities**. Differences between observed and predicted height velocities after 1 year of GH therapy in (a) ONH/SOD group and (b) non-ONH/SOD CGHD group were expressed in terms of Studentized residuals.

After one year on GH therapy, the two groups had similar changes in height SDS (p = 0.066). The ONH/SOD group continued to have a significantly higher height SDS, weight SDS, and BMI SDS after one year of GH therapy (all p < 0.001). However, height SDS corrected for family height genetics was significantly greater in the comparator group after the first year of GH treatment (Figure 1). Use of the prediction model for first-year growth response in children with idiopathic GHD showed that, although there was a slightly greater than predicted response in the ONH/SOD group versus the non-ONH/SOD CGHD group, there was no difference in actual height response between both groups, with Studentized residuals equivalent in both groups (Figure 2).

**Figure 3:**
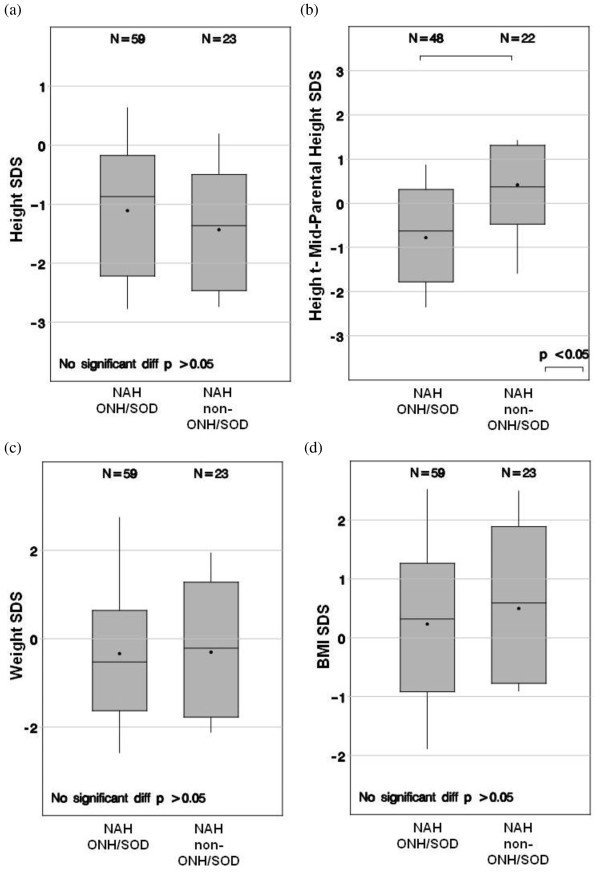
**Comparison between groups with ONH/SOD vs. non-ONH/SOD CGHD**. Comparison between groups with ONH/SOD vs. non-ONH/SOD CGHD of (a) height SDS, (b) height - MPH SDS, (c) weight SDS, and (d) BMI SDS at near-adult height.

NAH data were available for 59 subjects in the ONH/SOD group and 23 subjects in the non-ONH/SOD CGHD group (Table 3). The two groups had similar NAH at approximately -1 SDS (p = 0.430). The non-ONH/SOD CGHD attained a NAH that was closer to their mid-parental height (p < 0.05). The two groups had similar weight SDS and BMI SDS at time of NAH (Figure 3). At NAH, the two groups had the same prevalence of hypogonadism.

**Table 3:** Characteristics at Near-Adult Height

	ONH/SOD			Non-ONH/SOD			
	N	Median	10^th^, 90^th^ percentiles	N	Median	10^th^, 90^th^ percentiles	P-value
	*At GH Start*						
Gender, males	59	26 (44.1%)		23	10 (43.5%)		
Age (years)	59	5.64	2.14, 11.76	23	5.12	2.09, 12.04	0.546
Height SDS	59	-3.77	-5.51, -1.96	23	-4.97	-7.73, -2.98	< 0.001
Height - Mid-Parental Height SDS	48	-3.41	-5.85, -1.78	22	-3.30	-5.81, -0.91	0.658
Weight SDS	59	-2.23	-4.44, -0.72	23	-3.49	-5.92, -0.80	0.013
BMI SDS	59	-0.13	-2.02, 1.86	23	0.09	-1.64, 1.56	0.955
GH Dose (mg/kg/week)	59	0.21	0.14, 0.31	23	0.21	0.14, 0.43	0.996
	*At NAH*						
Age (years)	59	17.73	15.5, 20.5	23	17.30	15.3, 19.4	0.369
Height SDS	59	-0.87	-2.8, 0.6	23	-1.37	-2.7, 0.2	0.430
Δ Height SDS (Latest minus Start)	59	2.75	1.1, 4.4	23	3.37	1.6, 6.1	0.025
Height - Mid-Parental Height SDS	48	-0.63	-2.35, 0.87	22	0.37	-1.59, 1.43	0.004
Weight SDS	59	-0.53	-2.6, 2.7	23	-0.22	-2.1, 1.9	0.988
BMI SDS	59	0.32	-1.90, 2.50	23	0.59	-0.90, 2.50	0.581
Mean Total GH Dose (mg/kg/week)	59	0.19	0.13, 0.28	23	0.19	0.14, 0.25	0.722
Years on GH Treatment	59	11.15	6.69, 16.37	23	11.51	6.71, 14.69	0.996
Hypogonadism	59	23 (39.0%)		23	9 (39.1%)		

SDS, standard deviation score; GH, growth hormone

## Discussion

Although over half of the patients with ONH/SOD will develop an abnormality in their GH-IGF-I axis [2], little is known about their clinical presentations and auxological responses to GH, especially in comparison to those of similar patients with non-ONH/SOD CGHD.

In our study, the birth length and weight were significantly greater in the ONH/SOD group. It is unclear whether these differences can be fully explained by the increased mid-parental height seen in the ONH/SOD group or if there might be other disease-specific explanations. Some of this difference in mid-parental height may be due to the parents of those children with non-ONH/SOD CGHD also having the same gene defect causing short stature. At the time of diagnosis, the two groups had similar stimulated peak GH levels suggesting a comparable degree of GH deficiency.

The higher prevalence of pituitary hormone deficiencies seen in the ONH/SOD group compared to the non-ONH/SOD CGHD group is not surprising given the known association of ONH/SOD with hypopituitarism. However, our study found, at the time of GH initiation, for no obvious reason, a much higher prevalence of associated hormone deficiencies in the ONH/SOD group compared to the data reported in the NCGS study (hypothyroidism: 63.2% vs 27%, glucocorticoid deficiency: 52.2% vs 24%, and diabetes insipidus: 12.9% vs 5%) [4]. In a smaller subset who reached NAH, 39% of subjects in our cohort had hypogonadism while this data was not reported in the NCGS study.

At the time of GH initiation, the ONH/SOD group was significantly larger than the non-ONH/SOD CGHD group in all measures (height, weight, and BMI). The taller heights at diagnosis in the ONH/SOD group might relate to the associated ophthalmological manifestations of the condition leading to nystagmus and earlier referral. This group's greater genetic height potential might also be contributory. As for the higher weight and BMI, this may be associated with the intrinsic hypothalamic dysfunction seen in some patients with ONH/SOD causing hyperphagia.

The two groups responded similarly to one year of GH therapy with a comparable increase in height and similar to that predicted using the KIGS-derived prediction model for first-year growth in GH-treated children with idiopathic GHD. As a result, the ONH/SOD group continued to have significantly greater height, along with weight and BMI, than did the non-ONH/SOD CGHD group. Although we recognize the small size of the subsets from both groups that attained NAH and the inherent uncertainty of being able to draw firm conclusions as a result, the available data suggest that the two groups have similar height, weight, and BMI at NAH. With height outcomes in both groups within 1 SD of the mean (corrected for their respective mid-parental heights), this suggests that there is excellent adult height potential in children with congenital GH deficiency. Furthermore, these results suggest that long-term GH therapy can possibly prevent, minimize, and/or reverse the obesity that has been described in patients with ONH/SOD. Additional randomized prospective studies of GH therapy in patients with ONH/SOD are needed to investigate the effects of GH therapy specifically on obesity and body composition.

In summary, children with ONH/SOD have different presenting characteristics, but similar and normal final height responses to GH therapy compared to children with non-ONH/SOD CGHD. Because ONH/SOD is a major risk factor for CGHD and these patients may not be as short as those with non-ONH/SOD CGHD, it is important for clinicians to have a high index of suspicion and begin screening these patients for CGHD as early as possible. GH therapy may have additional benefits in this patient population as well with regard to body composition thus making early diagnosis and treatment even more important.

## Abbreviations

ONH: Optic nerve hypoplasia; SOD: septo-optic dysplasia; GH: growth hormone; NCGS: National Cooperative Growth Study; NAH: near-adult height; CGHD: congenital growth hormone deficiency; SDS: standard deviation score

## Competing interests

HK is a full-time employee of Pfizer Endocrine Care, KIGS/KIMS/ACROSTUDY, Sollentuna, Sweden. MG receives funding from Pfizer for institutional participation in KIGS (Pfizer International Growth Database); for research subject participation in the multi-center clinical trial: "A Four-Year Open-Label Multi-Center Randomized Two-Arm Study of Genotropin in Idiopathic Short Stature Patients: Comparing an Individualized, Target-Driven Treatment Regimen to Standard Dosing of Genotropin," and for serving as a member of national and international KIGS advisory boards.

## Authors' contributions

AV participated in the design of the study and drafted the manuscript. HK participated in the design of the study and performed the statistical analyses. CF and MB participated in the study's design and coordination. MG conceived of the study, and participated in its design and coordination. All authors read and approved the final manuscript.
